# A Case Report of follicular dendritic cell sarcoma

**DOI:** 10.3389/fonc.2026.1829976

**Published:** 2026-06-18

**Authors:** Lijiao Wang, Tingshu Jiang

**Affiliations:** 1 School of Clinical Medicine, Shandong Second Medical University, Weifang, Shandong, China; 2 Department of Respiratory and Critical Care Medicine, Yantai Yuhuangding Hospital Affiliated to Qingdao University, Yantai, Shandong, China

**Keywords:** diagnosis, follicular dendritic cell sarcoma, immunohistochemistry, PD-1 inhibitor, therapy

## Abstract

Follicular dendritic cell sarcoma (FDCS) is a rare malignant neoplasm originating from follicular dendritic cells. We present a case of advanced FDCS arising in the mediastinum with multiple metastases. The diagnostic complexity, aggressive clinical course, and integrative therapeutic approach involving immunotherapy and targeted therapy offer clinically relevant insights for managing this rare and challenging malignancy.

## 1 Case report

A 51-year-old woman presented with a 6-month history of cough, expectoration, and chest tightness, which had worsened over the past month, causing dyspnea after walking approximately 100 meters on level ground. She initially visited Laizhou People’s Hospital, where a chest CT scan revealed a mediastinal mass highly suspicious for malignancy and multiple enlarged lymph nodes. She was subsequently transferred to the Department of Integrated Traditional Chinese and Western Medicine at our hospital. She reported a 15-kg weight loss over the previous 3 months. Her medical history was notable for vitiligo lasting over 10 years, and story her mother had hepatocellular carcinoma. On admission, physical examination revealed coarse breath sounds in both lungs with occasional dry rales. She was initially diagnosed with a mediastinal tumor. Subsequent evaluations included tumor marker analysis on April 12, 2023, which demonstrated an elevated squamous cell carcinoma antigen level of 3.8 ng/mL. Ultrasonography performed the same day revealed mild enlargement of left cervical lymph nodes and marked enlargement of right cervical lymph nodes. Contrast-enhanced abdominal CT on April 12, 2023, showed mildly enlarged lymph nodes in the hepatogastric space, and a mediastinal mass. A contrast-enhanced chest CT scan on April 16, 2023, revealed multiple nodules and masses in the mediastinum and hepatogastric space, raising suspicion for Castleman disease (**Figure 1A**). Bronchoscopy on April 19, 2023, indicated the presence of a mediastinal mass. Transbronchial needle aspiration (TBNA) biopsy on April 21, 2023, yielded only scant fragmented lymphoid tissue within an inflammatory and hemorrhagic background, without definitive malignant features. Brush cytology also showed no tumor cells. A biopsy of the mediastinal mass obtained on April 27, 2023, revealed minimal lung tissue exhibiting focal hyaline degeneration of fibrous tissue and scattered lymphocyte infiltration. Following consultation with hematology, the patient was transferred to the hematology department for further evaluation. A whole-body 18F-FDG PET-CT scan on May 1, 2023, demonstrated multiple FDG-avid nodules and masses in the mediastinum and hepatogastric space, strongly suggesting Castleman disease (**Figure 2**), and multiple bilateral cervical lymph nodes, some demonstrating increased FDG uptake (possibly inflammatory in nature) (**Figure 2**). After consultation with respiratory medicine, medical thoracoscopic biopsy was deemed unsuitable, and thus, on May 9, 2023, a planned thoracoscopic exploration was converted to open thoracotomy to obtain tissue from the mediastinal mass and regional lymph nodes. The pathological specimen received on May 16, 2023, consisted of an irregular gray-white tissue fragment measuring 1.8 × 1.7 × 0.7 cm. The cut surface was color soft, varying from gray-white to gray-yellow. Histopathological analysis of hematoxylin and eosin (H&E)-stained sections is shown in **Figure 3**, while immunohistochemistry and *in situ* hybridization results *in situ* are summarized in **Table 1**. Representative immunohistochemical staining images are presented in **Figure 3**. Based on comprehensive imaging and histopathological findings (including immunohistochemistry), the patient was diagnosed with follicular dendritic cell sarcoma (FDCS) with metastatic involvement of the neck, mediastinum, and abdomen. A timeline of the clinical presentation, diagnostic workup, and disease course is summarized in **Figure 4**.

**Figure 1:**
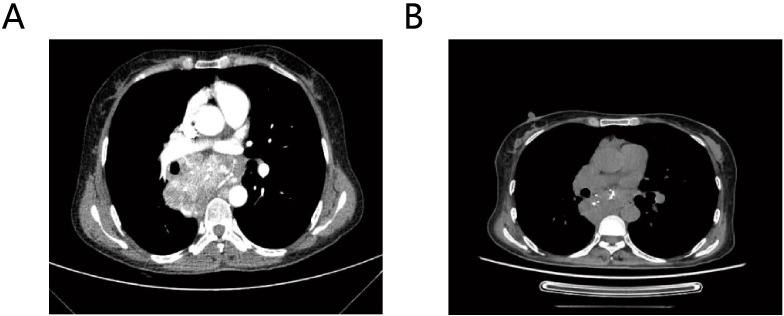
Contrast-enhanced and non-contrast chest computed tomography (CT). **(A)** (April 16, 2023): Contrast-enhanced chest CT showing multiple nodules and masses in the mediastinum and hepatogastric space, suggestive of possible Castleman disease. **(B)** (July 1, 2023): Non-contrast chest CT demonstrating a posterior mediastinal mass with a slight size reduction compared to the previous examination; other multiple nodules and masses in the mediastinum show mild interval regression in certain areas.

**Figure 2:**
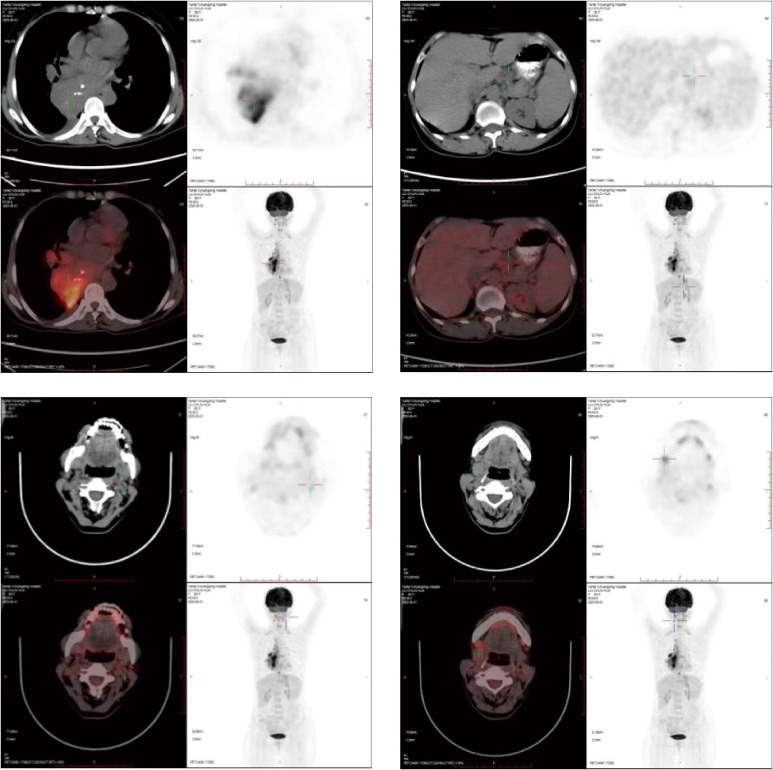
Whole-body 18F-FDG PET/CT imaging (May 1, 2023). 1. Multiple nodules and masses in the mediastinum and gastrohepatic space, with increased FDG uptake, suggestive of possible Castleman disease. 2. Multiple bilateral cervical lymph nodes, some exhibiting elevated FDG uptake, indicating possible inflammatory pathology.

**Figure 3:**
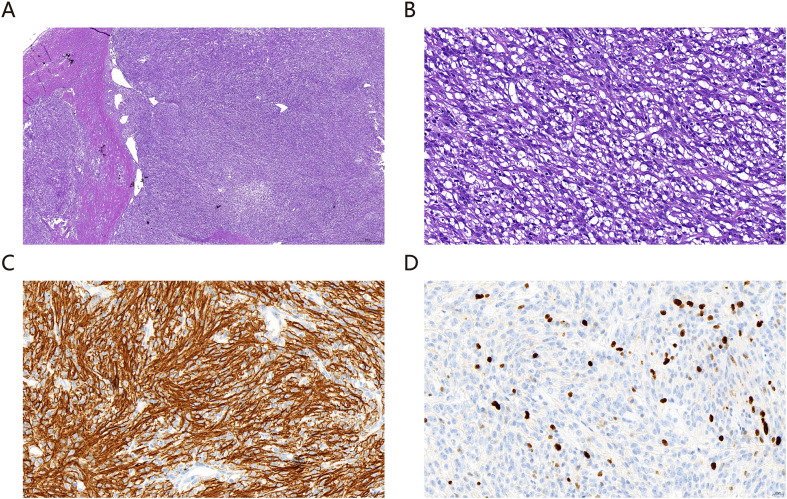
Histopathological findings of mediastinal mass biopsy and immunohistochemical analysis of mediastinal mass biopsy [**(A)** HE staining, ×4; **(B)** HE staining, ×400, **(C)** CD21, ×400; **(D)** Ki-67, ×400]. **(A, B)** Tumor cells exhibit spindle or oval morphology arranged in whorled, fascicular, and storiform patterns, with moderate amounts of eosinophilic cytoplasm and indistinct cell borders, resulting in a syncytium-like appearance. Nuclei are elongated with vesicular chromatin, thin nuclear membranes, and small but prominent nucleoli. A mild infiltrate of small lymphocytes is typically observed among the tumor cells. **(C)** CD21 staining demonstrates diffuse membranous positivity. **(D)** Ki-67 staining shows scattered positivity.

**Table 1:** Comprehensive immunohistochemical and *in situ* hybridization results.

Category	Markers	Results
In Situ Hybridization	EBER	Negative
IHC Positive Markers	Vim	Diffuse positive
	CD21	Positive
	D2-40	Positive
	EMA	Focal positive
	S-100	Focal positive
	SMA, Desmin	Focal positive
	PD-1	Scattered positive cells
	PD-L1 (SP263)	CPS ≈ 30
	Ki-67	~20% positive
IHC Negative Markers		
Epithelial Markers	CK, CK19, EGFR	Negative
Background Cell Markers	CD23	Negative
Solitary Fibrous Tumor Markers	STAT6	Negative
Melanocytic Markers	SOX-10	Negative
Mesenchymal Markers	ALK	Negative

**Figure 4:**
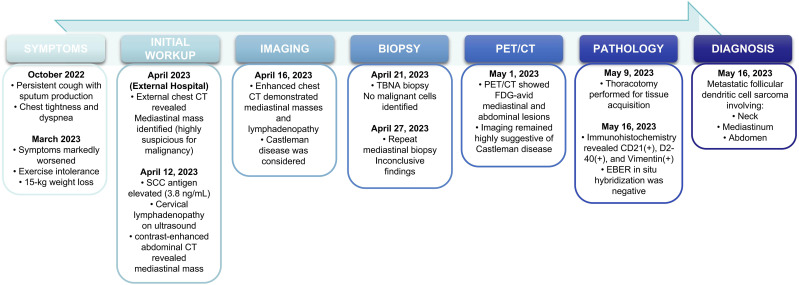
Timeline of clinical presentation, diagnostic workup, and disease course.

Treatment and Prognosis: After the diagnosis of advanced FDCS, first-line therapy was initiated using a combination of gemcitabine (1000 mg/m² on days 1 and 8) and docetaxel (100 mg/m² on day 8). The first treatment cycle commenced on May 17, 2023, with the day-8 dose administered on May 25, 2023. On June 11, 2023, she was admitted for combination therapy involving a PD-1 monoclonal antibody with gemcitabine and docetaxel. During outpatient intervals, she regularly received oral administration of a VEGFR inhibitor. Subsequently, on June 21, 2023, she was hospitalized for therapy incorporating PD-1 monoclonal antibody, gemcitabine, docetaxel, and the targeted agent pazopanib. A follow-up non-contrast chest CT scan on July 1, 2023, revealed a posterior mediastinal mass with a slight size reduction compared to previous imaging; however, multiple other mediastinal nodules and masses exhibited no significant change (**Figure 1B**). On July 10, 2023, the patient developed progressively worsening dyspnea. Her family declined invasive interventions, including endotracheal intubation or tracheostomy, and requested discharge against medical advice. Telephone follow-up confirmed that the patient died later the same day, resulting in an overall survival of approximately one month from diagnosis. This case report was approved by the Ethics Committee of Yantai Yuhuangding Hospital (Approval No.: K2026-037). Written informed consent was obtained from the patient’s family.

## 2 Discussion

FDCS is a mesenchymal neoplasm originating from follicular dendritic cells, characterized by low- to intermediate-grade malignant potential and first described by Monda et al. in 1986 (1). FDCS exhibits no significant gender predilection, with a median age at diagnosis of approximately 50 years. Clinical manifestations of FDCS are highly heterogeneous and largely nonspecific. Clinically, FDCS most commonly presents as a painless, slowly enlarging lymph node mass; however, extranodal involvement occurs frequently, with an incidence as high as 79.4%. Primary mediastinal involvement is an exceptionally rare manifestation of FDCS, comprising only 3.2% of extranodal FDCS cases. Systemic symptoms, although less common, may include fatigue, fever, and night sweats (2). Local symptoms primarily result from mechanical compression by the tumor mass. Mediastinal FDCS often presents with symptoms secondary to compression of adjacent structures such as the trachea, esophagus, or major vessels, causing cough, chest tightness, and dyspnea. These compressive symptoms align with the initial presentation of cough, expectoration, and chest tightness observed in our patient. FDCS typically follows an indolent clinical course, with a median survival of approximately 10 years for localized disease; nevertheless, nearly half of affected individuals eventually experience local recurrence or distant metastases. Common metastatic sites include the head and neck region and the thoracic, abdominal, and pelvic cavities (3, 4). Our patient presented with a primary mediastinal lesion accompanied by multiple cervical and abdominal metastases, consistent with advanced extranodal FDCS reported in the literature (1). Initial radiological findings strongly suggested Castleman disease; however, definitive diagnosis required histopathological examination and immunohistochemistry. This case underscores the diagnostic challenge of distinguishing FDCS from Castleman disease and highlights the importance of comprehensive histopathological evaluation and immunohistochemical analysis. Therefore, the diagnosis depended on exclusion criteria supported by a thorough diagnostic assessment. The diagnosis rests upon three key findings: (1) spindle or oval-shaped tumor cells arranged in whorled, fascicular, and storiform patterns, with moderate amounts of eosinophilic cytoplasm and indistinct borders producing a syncytium-like appearance; elongated nuclei containing vesicular chromatin, thin nuclear membranes, and small, prominent nucleoli; and a mild infiltrate of small lymphocytes among tumor cells; (2) tumor cells exhibiting positivity for CD21, a defining marker for FDCS; and (3) systematic exclusion of other spindle cell neoplasms and inflammatory conditions through extensive immunohistochemical analysis. A comprehensive differential diagnosis is presented in **Table 2**.

**Table 2:** Comprehensive differential diagnosis of follicular dendritic cell sarcoma (FDCS).

Diagnosis entity	Key positive IHC markers	Present case results and rationale for exclusion	Conclusion
FDCS (Present Case)	CD21, CD23, CD35, D2-40, TTF-1, and synaptophysin	CD21(+), D2-40(+)	Definitively diagnosed
Castleman Disease	Follicular dendritic cell hyperplasia	CD21(+),D2-40(+)	Ruled out
Inflammatory Pseudotumor/Reactive Lesions	No specific immunophenotypic markers	CD21(+),D2-40(+)	Ruled out
Gastrointestinal Stromal Tumor (GIST)	KIT (CD117), DOG1, CD34	All negative	Ruled out
Inflammatory Myofibroblastic Tumor (IMT)	ALK, SMA, Calponin	ALK negative; focal SMA positivity is a nonspecific feature	Ruled out
EBV+ iFDCS	Essential: EBER(diffuse) Supportive: FDC markers(e.g.CD21, CD23, CD35)	EBER (-)	Ruled out
Interdigitating Dendritic Cell Sarcoma (IDCS)	S-100	All negative	Ruled out
Malignant Melanoma	S-100, SOX10, HMB45, Melan-A	All negative	Ruled out
Lymphoma (B-cell, T-cell, ALCL)	CD45, CD20, CD3, CD30	All negative	Ruled out
Thymoma	Cytokeratin, TdT (immature T cells)	All negative	Ruled out
Langerhans cell neoplasm	CD1a, Langerin, S-100	All negative	Ruled out
Histiocytic Sarcoma	CD68, CD163, Lysozyme	All negative	Ruled out
Poorly Differentiated/Undifferentiated Carcinoma	Pan-cytokeratin, p40/p63, EMA	All negative	Ruled out
Solitary Fibrous Tumor (SFT)	STAT6, CD34	All negative	Ruled out
Ectopic Meningioma	EMA, PR	All negative	Ruled out

The pathogenesis of FDCS remains unclear. Recognized associated factors include Castleman disease, autoimmune disorders, and Epstein-Barr virus (EBV) infection (2, 5, 6). Our patient had a longstanding history of vitiligo (>10 years). Multiple studies have demonstrated a link between FDCS and autoimmune diseases such as paraneoplastic pemphigus and myasthenia gravis (2, 5), suggesting that immune dysregulation, possibly mediated by aberrant inflammatory activation or impaired immune surveillance, may play a role in FDCS pathogenesis. This case contributes additional clinical evidence and valuable insights for future etiological research.

Imaging plays a crucial role in the initial assessment and staging of FDCS but often lacks diagnostic specificity. Contrast-enhanced CT typically reveals a heterogeneously enhancing primary mass with occasional central necrosis. In our case, contrast-enhanced chest CT showed multiple nodules and masses in the mediastinum and hepatogastric space, raising suspicion for Castleman disease. 18F-FDG PET/CT demonstrated multiple hypermetabolic lesions in the mediastinum, neck, and hepatogastric space, consistent with imaging characteristics of metastatic FDCS (7). The presence of mediastinal metastasis usually indicates advanced disease and suggests a poor prognosis. Given these characteristic imaging findings, FDCS should be considered in the differential diagnosis of patients presenting with unexplained mediastinal masses and multiple hypermetabolic nodules, particularly those with immune-related comorbidities or systemic symptoms. Such suspicion warrants timely biopsy for definitive pathological confirmation.

FDCS is a rare malignant tumor originating from follicular dendritic cells located within lymph node germinal centers and is classified as a mesenchymal-derived dendritic cell tumor. According to the 2022 World Health Organization (WHO) classification, FDCS is categorized as a stromal-derived neoplasm of lymphoid origin (8). Histologically, conventional FDCS features spindle-shaped or ovoid tumor cells arranged in fascicular, whorled, or storiform patterns, typically accompanied by scattered small lymphocytes. High-grade FDCS demonstrates pronounced nuclear atypia, extensive necrosis, and increased mitotic activity, associated with enhanced aggressiveness and metastatic potential (8, 9). Immunohistochemically, FDCS commonly expresses follicular dendritic cell markers, including CD21, CD23, and CD35, with occasional while aberrant expression of D2-40, TTF-1, and synaptophysin. Tumor cells are typically negative for epithelial and myogenic markers. At the molecular level, abnormal activation of the NF-κB signaling pathway, along with inactivation of cell-cycle regulatory genes (e.g., CDKN2A, RB1) and mutations in TP53, contributes to uncontrolled proliferation and malignant transformation (10). Furthermore, the tumor microenvironment exhibits immune suppression and abnormal angiogenesis, facilitating tumor progression, recurrence, and metastasis (8, 10, 11). A significant proportion of FDCS cases demonstrate elevated PD-L1 expression, suggesting immune evasion and providing a rationale for immunotherapeutic interventions (8–10). In the present case, immunohistochemical analysis revealed positivity for CD21 and D2–40 and negativity for CD23, consistent with FDCS diagnosis. Moreover, PD-L1 expression assessed using the SP263 assay revealed a combined positive score (CPS) of approximately 30. Since PD-L1 positivity is reported in 50%-80% of FDCS cases (6, 11), this finding supports the biological basis for including immune checkpoint inhibitors in treatment strategies.

To date, standardized therapeutic guidelines for FDCS have not been established due to its rarity and variable clinical behavior. Current management generally involves multimodal strategies incorporating surgery, chemotherapy, radiotherapy, targeted therapy, and recently, immunotherapy. Surgical resection remains the primary treatment modality whenever feasible; however, local recurrence and distant metastasis frequently occur, particularly in patients with extranodal disease, who typically experience poorer outcomes and often require multimodal treatment (12). Responses to systemic therapies vary, as documented by several case reports. For instance, Riaz et al. reported the first pediatric case of parotid FDCS treated with CCABE chemotherapy (cisplatin, cyclophosphamide, bleomycin, etoposide, doxorubicin) combined with 40 Gy external beam radiotherapy, achieving complete remission of the primary lesion and initial regression of pulmonary metastases. Importantly, this regimen remained effective when pulmonary recurrence occurred (13). Chen et al. similarly described a patient with Epstein-Barr virus (EBV)-positive conventional FDCS treated with surgical resection followed by two cycles of CHOP chemotherapy, resulting in stable residual lymphadenopathy without further progression (14). Favorable outcomes were also reported in hepatic FDCS treated with surgery and CHOP chemotherapy, showing reduced FDG uptake and increased tumor necrosis despite minimal tumor size changes (15). Among systemic treatments, gemcitabine-based regimens demonstrate notable activity. Jain et al. reported an overall response rate of 80% with gemcitabine-taxane chemotherapy in FDCS (16). Likewise, durable partial responses were observed in two patients with metastatic hepatic FDCS treated with gemcitabine and docetaxel (17). These findings support the use of gemcitabine-taxane combinations for advanced FDCS Radiotherapy also plays an essential role in disease control, especially postoperatively. Wang et al. described a case involving a middle-aged woman with mediastinal FDCS and paraneoplastic pemphigus who underwent thoracoscopic resection followed by corticosteroid therapy, antifungal treatment, and radiotherapy, achieving significant clinical improvement at one -year follow-up (9). Similarly, Eun et al. described a patient who achieved a two-year disease-free interval following adjuvant radiotherapy (18). Furthermore, Chen et al. presented the first case of tonsillar FDCS treated with CHOP chemotherapy, radiotherapy, and pembrolizumab, achieving prolonged disease-free survival (10). The increasing recognition of immune checkpoint pathways in FDCS has stimulated interest in immunotherapy; however, clinical outcomes remain inconsistent. Resnick et al. reported a recurrent inflammatory pseudotumor-like FDCS treated with surgical resection followed by ipilimumab and nivolumab therapy. Although disease progression occurred during immunotherapy, subsequent salvage surgery achieved durable disease control (19). These findings suggest that immunotherapy may benefit selected patients but is likely best incorporated into comprehensive treatment strategies rather than as monotherapy. Other reports further emphasize the diversity of treatment responses in FDCS. Hu et al. described a mediastinal FDCS with thoracic vertebral invasion and iliac metastasis successfully treated with surgical resection and body gamma-knife radiotherapy, achieving complete pathological remission of the iliac lesion and stable disease for 15 months (12). Kashyap et al. detailed a patient with metastatic high-grade FDCS who underwent pneumonectomy, cytoreductive surgery, CHOP chemotherapy, paclitaxel-gemcitabine chemotherapy, and maintenance lenalidomide, resulting in nearly seven years of disease control with only one manageable recurrence (8). In contrast, EBV-positive inflammatory pseudotumor-like FDCS generally demonstrates more favorable outcomes. Bai et al. reported three patients with EBV-positive IPT-like FDCS who underwent complete surgical resection; all patients were alive at last follow-up, and recurrence in one hepatic case was successfully managed with ablation therapy (20). Similarly, Chen et al. analyzed 16 patients with hepatosplenic EBV-positive IPT-like FDCS treated with complete surgical excision and found no recurrence or metastasis during 1–60 months of follow-up, indicating an excellent prognosis (21). However, treatment resistance remains a significant issue in advanced disease. Feng et al. reported axillary FDCS with postoperative recurrence and bone metastasis refractory to multiple treatments, including repeated surgery, radiofrequency ablation, CHOP chemotherapy, gemcitabine-docetaxel, and albumin-bound paclitaxel. Subsequent treatment with apatinib achieved stable disease, yielding a progression-free survival of 10 months (11). In the present case, extensive metastatic involvement of the neck, mediastinum, and abdomen precluded surgical intervention. Consequently, a multimodal regimen comprising a PD-1 inhibitor combined with gemcitabine, docetaxel, and pazopanib was administered, reflecting emerging treatment paradigms integrating chemotherapy, targeted therapy, and immunotherapy for advanced soft tissue malignancies (22, 23). Despite an initial radiographic tumor reduction, rapid disease progression ensued, leading to the patient’s death shortly after diagnosis. This unfavorable outcome highlights significant therapeutic challenges associated with advanced FDCS, potentially attributable to marked tumor heterogeneity, inadequate antitumor immune activation despite PD-L1 expression, and intrinsic or acquired resistance mechanisms. Therefore, further studies are needed to elucidate the molecular landscape of FDCS and to develop personalized therapies guided by comprehensive molecular profiling.

Prognosis in FDCS depends on multiple clinicopathological factors. Established adverse prognostic indicators include tumor size exceeding 6 cm, intra-abdominal location, coagulative necrosis, high mitotic count (>5 per 10 high-power fields), and marked nuclear atypia (1). Local recurrence rates range from 28% to 50%, and metastasis risk is significantly higher in extranodal FDCS (>20%) compared to nodal FDCS (~10%). The two-year survival rate for advanced-stage FDCS is approximately 42% (1, 2). Mediastinal metastasis generally indicates advanced disease and portends a poor prognosis, as illustrated by the dismal clinical outcome of our patient despite comprehensive therapy. This aligns with existing reports indicating poor prognoses in advanced FDCS. Recent genomic and transcriptomic studies have revealed unique molecular profiles in FDCS involving dysregulated NF-κB signaling and cell cycle control pathways, thus providing potential targets for precision therapies (24). Future research should prioritize large-scale, multicenter studies to further clarify FDCS molecular mechanisms. Concurrently, clinical trials evaluating targeted and immunotherapies are essential for optimizing individualized treatments and achieving meaningful survival benefits.

## Data Availability

The original contributions presented in the study are included in the article/supplementary material. Further inquiries can be directed to the corresponding author.
